# Complement regulation in ovine lymph nodes during early pregnancy

**DOI:** 10.3892/etm.2021.11089

**Published:** 2021-12-23

**Authors:** Leying Zhang, Lidong Cao, Pengfei Feng, Xu Han, Ling Yang

**Affiliations:** Department of Animal Science, School of Life Sciences and Food Engineering, Hebei University of Engineering, Handan, Hebei 056038, P.R. China

**Keywords:** lymph node, pregnancy, complement component, sheep

## Abstract

A fetus changes immune responses in the uterus and the maternal immune system, and lymph nodes are associated with regulating maternal adaptive immunity. Complement activation is associated with abnormal pregnancy in mice and humans. The aim of the present study was to explore the expression levels of complement components in maternal lymph nodes during early pregnancy in sheep. Maternal inguinal lymph nodes were sampled on day 16 of the estrous cycle, and days 13, 16 and 25 of gestation in ewes. Reverse transcription-quantitative PCR, western blotting and immunohistochemical analyses were used to detect the expression levels of complement components C1q, C1r, C1s, C2, C3, C4a, C5b and C9 in the lymph nodes. The results revealed that the protein and mRNA levels of C1q, C1s and C5b were enhanced during early pregnancy, and that C1r and C4a were upregulated at day 25 of pregnancy. The mRNA and protein levels of C2 and C9 peaked at day 16 of pregnancy, but C3 was decreased at day 25 of pregnancy. C3 protein was located in the subcapsular sinuses and lymph sinuses of the maternal lymph node. In summary, the present study detected changes in the expression levels of complement components in maternal lymph nodes, which may be associated with maternal immune regulation during early pregnancy in sheep.

## Introduction

During pregnancy, the fetus not only evades but also provokes immune responses in the uterus, maternal peripheral tissues and immune system, which are essential for the success of pregnancy (1). Lymph nodes are secondary lymphoid organs that participate in inducing and regulating adaptive immunity (2). Pregnancy induces an increase in the weights of lumbar, renal and inguinal lymph nodes in mice (3), and the weights of iliac lymph nodes also increase progressively during pregnancy in rats (4).

It has been reported that early pregnancy induces increases in the expression levels of progesterone (P4) receptor, P4-induced blocking factor (5), cyclooxygenase 1 (COX-1), COX-2, prostaglandin E synthase, Aldo-keto reductase family 1 member B1(6), interleukin (IL)-5 and IL-10, but TNF-β and IL-2 are decreased in the maternal lymph nodes during early pregnancy in sheep (7). Furthermore, there are increases in the protein expression levels of melatonin receptor 1, cluster of differentiation 4, signal transducer and activator of transcription 1, 2',5'-oligoadenylate synthetase, myxovirus resistance protein 1, C-X-C motif chemokine 10 and gonadotropin releasing hormone and its receptor in the maternal lymph nodes during early pregnancy in sheep (8-10). Early pregnancy induces changes in maternal lymph nodes, which may be associated with maternal immune tolerance to paternal alloantigens.

The complement system plays important roles in innate immune defense, which are associated with shaping adaptive immune responses (11). The major effector fragments of the complement system that are associated with the innate and adaptive immune systems include complement components C1, C3, C5 and C9, and the C1 complex (C1qC1r2s2), while C2 and C4 are implicated in C3 convertase formation (12).

Complement activation can promote inflammation and facilitate macrophage phagocytosis to placenta-derived particles and apoptotic cells, and the complement system also serves as a regulator of complex tolerance in healthy pregnancy (13). Complement components are located in placental tissue, and are associated with protecting the semiallogenic conceptus against the maternal immune system in humans (14). However, a complement-mediated immune attack against semiallogeneic fetal tissues can occur, which leads to adverse pregnancy outcomes in humans and animals (15). Early pregnancy regulates expression of complement components in the liver and thymus in the ovine (16,17). At present, it is unclear whether the expression of complement components is changed in maternal lymph nodes during early pregnancy in sheep.

During early gestation in ruminants, the trophectoderm of the conceptus secretes interferon-tau (IFNT), which participates in the maternal recognition of pregnancy and prevents luteolysis (18). IFNT increases the expression of interferon stimulated genes (ISGs) in the corpus luteum (CL) in an endocrine manner (19), and enhances the expression of ISGs in immune organs, including bone marrow (20), the thymus (21), the spleen (22,23) and lymph nodes (6,9) during early pregnancy in sheep.

It is hypothesized that early pregnancy affects the expression of complement components in ovine lymph nodes. The objective of the present study was to explore the expression of the complement components C1q, C1r, C1s, C2, C3, C4a, C5b and C9 in maternal lymph nodes, which may be helpful for understanding the immune regulation of maternal lymph nodes during early pregnancy in sheep.

## Materials and methods

### Animals and experimental design

A total of 24 small-tail Han ewes (*Ovis aries*) ~18 months old and average weight of 41 kg were observed daily for estrus in the presence of vasectomized rams at a farm in Handan, China. The ewes were housed under a condition of average atmospheric pressure of 1,027 hectopascals, a 11 h light/13 h dark cycle, temperature of 3-18˚C and free access to food and water. The ewes were randomly divided into four groups (n=6 for each group), and at estrus (day 0), the animals were mated to either intact or vasectomized rams. The females were necropsied on days 13, 16 and 25 of pregnancy, and day 16 of the estrous cycle as described previously (9). Euthanasia of the ewes was performed by an experienced person to cut both the carotid arteries and jugular veins to bleed out the animals after electrical stunning. The uterus was flushed, and pregnancy was confirmed by the presence of a normal conceptus in the uterine lumen flushing. Transverse pieces of the inguinal lymph nodes were collected immediately after slaughter and snap-frozen in liquid nitrogen (-196˚C) until subsequent mRNA and protein analyses. In addition, longitudinal cross sections of the lymph nodes were cut into pieces (0.3 cm^3^), and fixed with fresh 4% buffered paraformaldehyde for 12 h at room temperature for subsequent immunohistochemical analysis. The study protocol was reviewed and approved by the Hebei University of Engineering Animal Care and Use Committee (approval no. 2019-017), and humane animal care and handling procedures were followed throughout the experiment.

### RNA extraction and reverse transcription-quantitative PCR assay

The samples (transverse pieces of the inguinal lymph nodes) were crushed in liquid nitrogen, and total RNA was isolated using TRNzol Universal Reagent (DP424; Tiangen Biotech Co., Ltd.) according to the manufacturer's recommendations. A FastQuant RT kit with gDNase (cat. no. KR106; Tiangen Biotech Co., Ltd.) was used to remove genomic DNA and synthesize cDNA according to the manufacturer's instructions. The specific primers (Table I) were designed based on the sequences in the NCBI database (http://www.ncbi.nlm.nih.gov/) for the ovine C1q, C1r, C1s, C2, C3, C4a, C5b and C9 genes, and were synthesized by Shanghai Sangon Biotech Co., Ltd. Primer matrix experiments were performed to determine the optimal primer concentrations. The amplification efficiencies of the primer sequences were evaluated before quantification, and were in an acceptable range (between 0.9 and 1.1). A CFX96 real-time PCR system (Bio-Rad Laboratories, Inc.) was used for quantitative PCR in a total volume of 20 µl in triplicate using a SuperReal PreMix Plus kit (Tiangen Biotech Co., Ltd.). Cycling conditions of PCR included an initial denaturation at 95˚C for 10 min followed by 40 cycles of denaturation (95˚C for 10 sec), annealing (60-62˚C for 20 sec) and extension (72˚C for 25 sec) followed by one cycle of final extension (72˚C for 7 min). The annealing temperatures were 60˚C for *C1q*, *C1s*, *C2*, *C3* and *C9*, or 62˚C for *C1r*, *C4a* and *C5b*. Mean threshold cycle values (Cq) for the target genes and CT values for the reference gene (*GAPDH*) of each sample were calculated from triplicate wells, and the relative transcript abundances for the target genes were calculated using the 2^-ΔΔCq^ analysis method (24). Mean Cq values of estrous cycle of day 16 were used to normalize the relative levels of mRNA transcripts.

### Western blotting

The samples (transverse pieces of the inguinal lymph nodes) were homogenized using RIPA lysis buffer (Tiangen Biotech Co., Ltd.) to isolate total protein, and a BCA protein assay kit (Tiangen Biotech Co., Ltd.) was used to determine the concentrations of the total proteins. Total protein samples (10 µg/lane) were separated by 12% SDS-PAGE gel, and transferred electrophoretically to polyvinylidene fluoride membranes (MilliporeSigma). The membranes were blocked with 5% (w/v) skim milk powder at 4˚C overnight, and then incubated with a mouse anti-C1q monoclonal antibody (cat. no. sc-53544), a mouse anti-C1r monoclonal antibody (cat. no. sc-514105), a mouse anti-C1s monoclonal antibody (cat. no. sc-365273), a mouse anti-C2 monoclonal antibody (cat. no. sc-373809), a mouse anti-C3 monoclonal antibody (cat. no. sc-28294), a mouse anti-C4a monoclonal antibody (cat. no. sc-271181), a mouse anti-C5b monoclonal antibody (cat. no. sc-398247) and a mouse anti-C9 monoclonal antibody (cat. no. sc-390000) (all 1:1,000; Santa Cruz Biotechnology, Inc.) at 4˚C overnight. After washing, the membranes were incubated with horseradish peroxidase (HRP) conjugated anti-mouse secondary antibody at a 1:2,000 dilution (cat. no. BL001A; Biosharp Life Sciences). The blots were detected using a pro-light HRP chemiluminescence kit (Tiangen Biotech Co., Ltd.). Densitometric analysis of the relative intensities of the blots was performed using a Quantity One v452 (Bio-Rad Laboratories, Inc.), and normalized to a reference protein (GAPDH) using an anti-GAPDH antibody (1:1,000; cat. no. sc-47724; Santa Cruz Biotechnology, Inc.).

### Immunohistochemical analysis

The paraffin-embedded sections (5-µm thick) were deparaffinized and rehydrated in a series of xylene and ethanol. Some sections (transverse sections of the inguinal lymph nodes) were stained by hematoxylin for 30 sec and eosin for 20 sec at room temperature before antigen retrieval and staining with antibodies. The sections were boiled in citrate solution for 12 min at 100˚C for antigen retrieval, washed with phosphate-buffered saline and then treated with 3% hydrogen peroxide to remove endogenous peroxidase activity. Blocking non-specific binding site in the sections was performed using 5% goat serum for 1 h at room temperature, and then incubated with the mouse anti-C3 monoclonal antibody (cat. no. sc-28294; Santa Cruz Biotechnology, Inc.) at a final dilution of 1:200 at 4˚C overnight. The tissue sections were further treated with the secondary antibody (cat. no. BL001A; Biosharp Life Sciences) for 45 min at room temperature. For negative control sections, the primary antibody was replaced with antiserum-specific isotype at the same dilution. A DAB kit (Enhanced HRP-DAB Chromogenic kit; Tiangen Biotech Co., Ltd.) was used to detect the specific binding sites according to the manufacturer's instructions. Digital images (magnification, x400) were captured using a light microscope (Nikon Eclipse E800; Nikon Corporation) with a DP12 digital camera. The intensities of staining digital images were examined independently by four observers. The immunostaining intensities of the samples from different ewes (n=6 for each group) were analyzed through the images in a blind manner. Staining intensities for C3 protein were calculated by assigning an immunoreactive intensity on a scale of 0 to 3, as described previously (25). The staining intensity was as follows: 0=negative; 1+=weak; 2+=strong.

### Statistical analysis

Statistical analysis was performed using least-squares ANOVA in mixed and general linear model procedures of the Statistical Analysis System v9.2 (SAS Institute, Inc.). Day and status (cyclic or pregnant), and interaction between day and status on expression of mRNA and protein were tested using repeated measure for multivariate analysis of variance. Numerical data were presented as least squares means with standard errors, and comparison of means was tested by Tukey HSD test. P<0.05 was considered to indicate a statistically significant difference.

## Results

### Relative expression levels of C1q, C1r, C1s, C2, C3, C4a, C5b and C9 mRNA in lymph nodes

**Figure 1:**
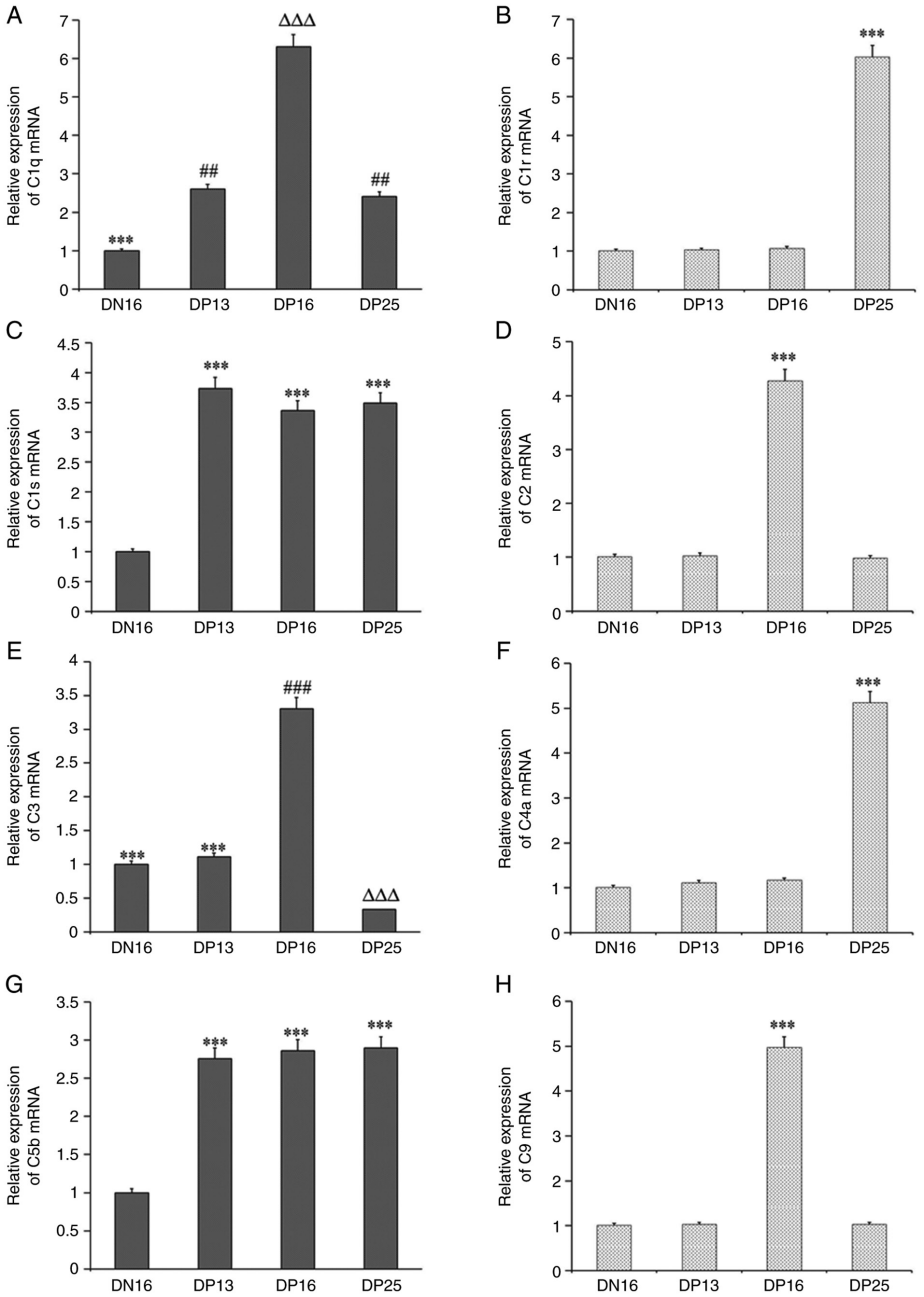
Relative expression values of *C1q, C1r, C1s, C2, C3, C4, C5* and *C9* mRNAs in the lymph nodes from the non-pregnant and pregnant ewes measured by reverse transcription-quantitative PCR. (A) Relative expression of C1q mRNA. ^***^P<0.001. DN16 vs. DP13, DP16 and DP25; ^##^P<0.01. DP13 and DP25 vs. DN16 and DP16; ^∆∆∆^P<0.001. DP16 vs. DN16, DP13 and DP25. (B) Relative expression of C1r mRNA. ^***^P<0.001. DP25 vs. DN16, DP13 and DP16. (C) Relative expression of C1s mRNA. ^***^P<0.001. DP13, DP16 and DP25 vs. DN16. (D) Relative expression of C2 mRNA. ^***^P<0.001. DP16 vs. DN16, DP13 and DP25. (E) Relative expression of C3 mRNA. ^***^P<0.001. DN16 and DP13 vs. DP16 and DP25; ^###^P<0.001. DP16 vs. DN16, DP13 and DP25; ^∆∆∆^P<0.001. DP25 vs. DN16, DP13 and DP16; (F) Relative expression of C4a mRNA. ^***^P<0.001. DP25 vs. DN16, DP13 and DP16. (G) Relative expression of C5b mRNA. ^***^P<0.001. DP13, DP16 and DP25 vs. DN16. (H) Relative expression of C9 mRNA. ^***^P<0.001. DP16 vs. DN16, DP13 and DP25. DN16, day 16 of the estrous cycle; DP13, day 13 of pregnancy; DP16, day 16 of pregnancy; DP25, day 25 of pregnancy.

The relative expression levels of *C1q, C1s* and *C5b* mRNA were significantly higher during pregnancy compared with day 16 of the estrous cycle (P<0.05), and the level of *C1q* mRNA was the highest at day 16 of pregnancy among the four groups (Fig. 1). The relative expression levels of C1r and C4a mRNAs were increased at day 25 of pregnancy compared with those at day 16 of the estrous cycle, and at days 13 and 16 of pregnancy (P<0.05). The levels of *C2* and *C9* mRNAs were the highest at day 16 of pregnancy among the four groups (P<0.05). In addition, the *C3* mRNA level was the highest at day 16 of pregnancy, and was the lowest at day 25 of pregnancy among the four groups (P<0.05).

### Expression levels of C1q, C1r, C1s, C2, C3, C4a, C5b and C9 proteins in lymph nodes

**Figure 2:**
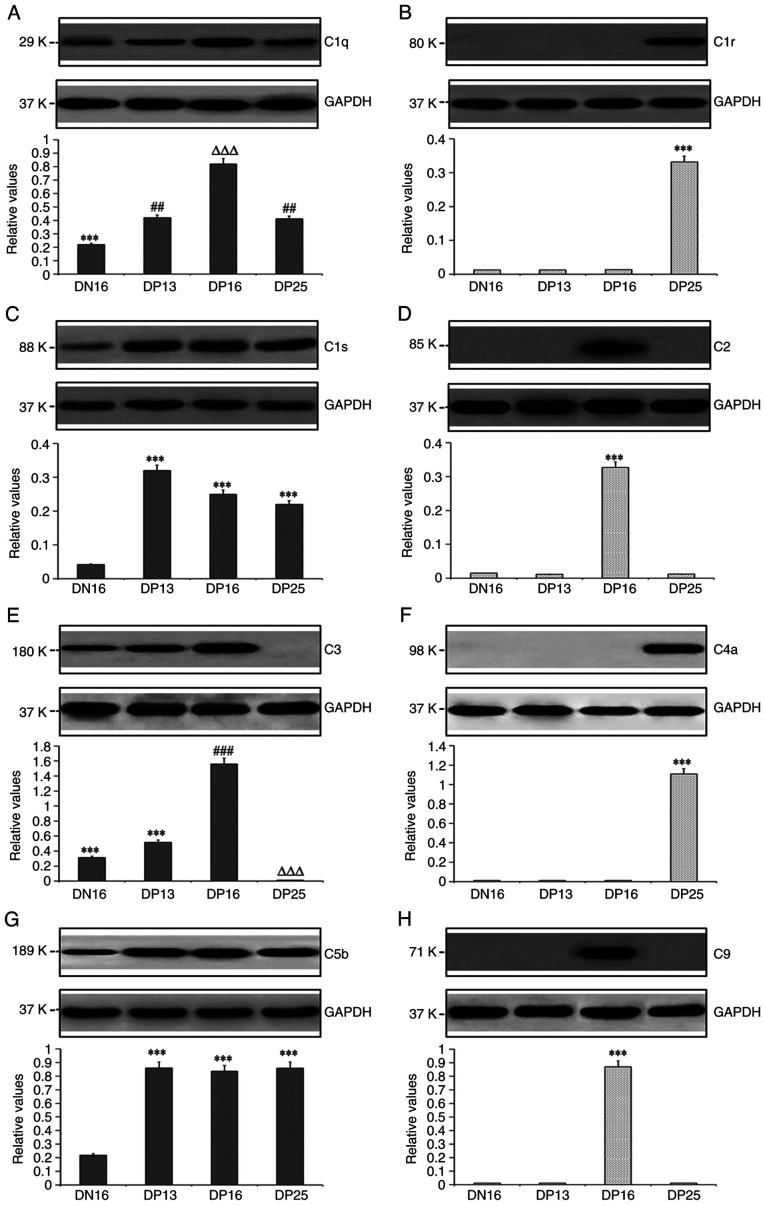
Expression levels of C1q, C1r, C1s, C2, C3, C4, C5 and C9 proteins in the lymph nodes from the non-pregnant and pregnant ewes analyzed using western blotting. (A) Relative expression of C1q protein. ^***^P<0.001. DN16 vs. DP13, DP16 and DP25; ^##^P<0.01. DP13 and DP25 vs. DN16 and DP16; ^∆∆∆^P<0.001. DP16 vs. DN16, DP13 and DP25. (B) Relative expression of C1r protein. ^***^P<0.001. DP25 vs. DN16, DP13 and DP16. (C) Relative expression of C1s protein. ^***^P<0.001. DP13, DP16 and DP25 vs. DN16. (D) Relative expression of C2 protein. ^***^P<0.001. DP16 vs. DN16, DP13 and DP25. (E) Relative expression of C3 protein. ^***^P<0.001. DN16 and DP13 vs. DP16 and DP25; ^###^P<0.001. DP16 vs. DN16, DP13 and DP25; ^∆∆∆^P<0.001. DP25 vs. DN16, DP13 and DP16; (F) Relative expression of C4a protein. ^***^P<0.001. DP25 vs. DN16, DP13 and DP16. (G) Relative expression of C5b protein. ^***^P<0.001. DP13, DP16 and DP25 vs. DN16. (H) Relative expression of C9 protein. ^***^P<0.001. DP16 vs. DN16, DP13 and DP25. DN16, day 16 of the estrous cycle; DP13, day 13 of pregnancy; DP16, day 16 of pregnancy; DP25, day 25 of pregnancy.

Western blotting indicated that there was almost no expression of C1s protein on day 16 of the estrous cycle, but pregnancy induced the expression of C1q, C1s and C5b proteins in lymph nodes compared with day 16 of the estrous cycle (P<0.05; Fig. 2). In addition, the C1q protein level was the highest at day 16 of pregnancy, and the C1s protein level was the highest at day 13 of pregnancy among the four groups (P<0.05). There was very low expression of C1r and C4a proteins at day 16 of the estrous cycle, and days 13 and 16 of pregnancy, but the expression levels of C1r and C4a proteins were significantly upregulated at day 25 of pregnancy compared with the other three groups (P<0.05). C2 and C9 proteins were highly expressed at day 16 of pregnancy, but their expression levels were very low at day 16 of the estrous cycle, and days 13 and 25 of pregnancy. Furthermore, the C3 protein level at day 16 of pregnancy was the highest among the four groups, and was very low at day 25 of pregnancy (P<0.05).

### Immunohistochemistry for C3 protein in the lymph nodes

**Figure 3:**
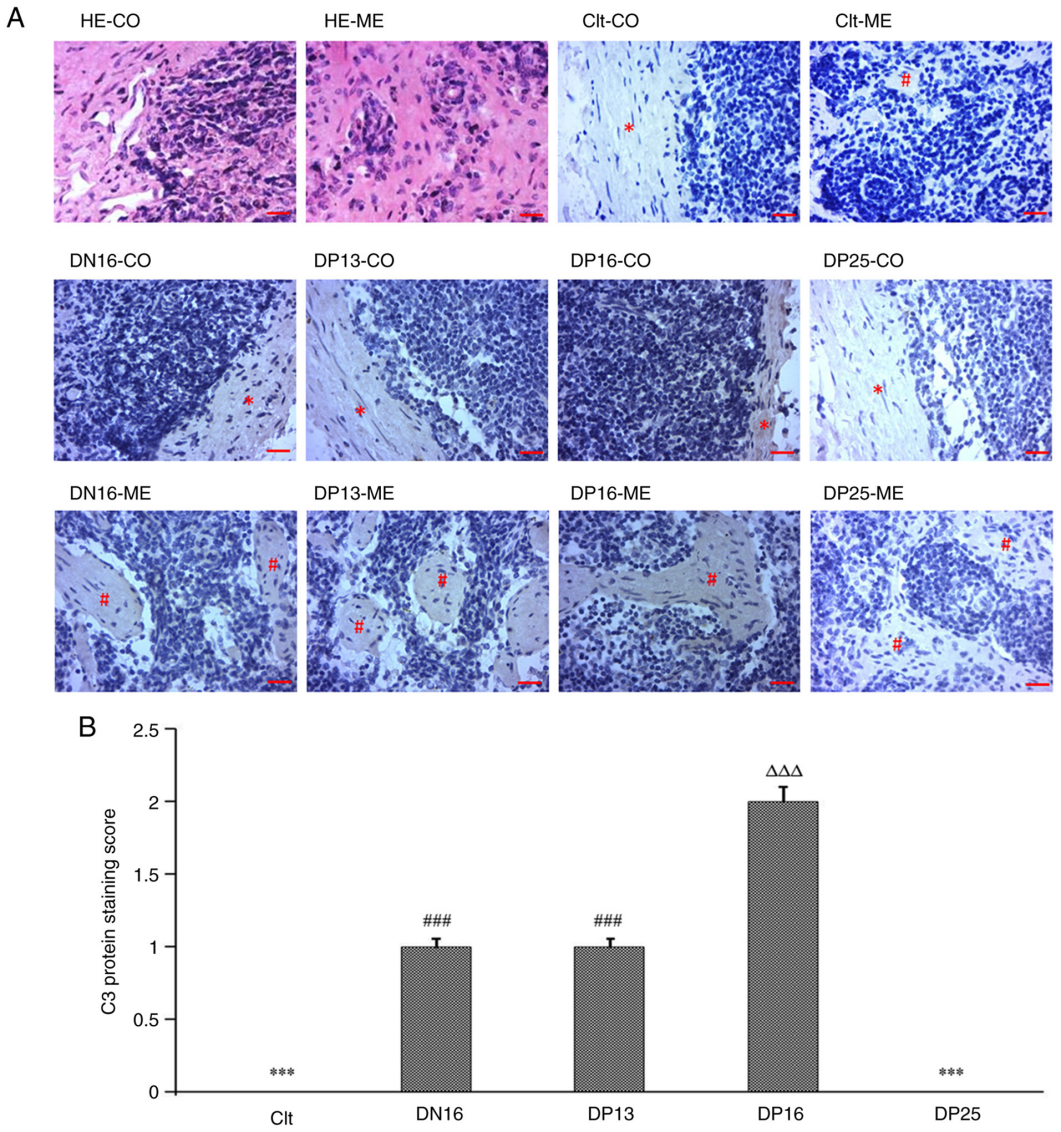
Immunohistochemical localization of C3 protein in the lymph nodes from non-pregnant and pregnant ewes. (A) Immunohistochemical localization of C3 protein. Lymph node is divided into the CO and the ME. Scale bar=20 µm. (B) Staining intensities for C3 protein. ^***^P<0.001. Clt and DP25 vs. DN16, DP13 and DP16; ^###^P<0.001. DN16 and DP13 vs. Clt, DP16 and DP25; ^∆∆∆^P<0.001. DP16 vs. Clt, DN16, DP13 and DP25. CO, cortex; ME, medulla; HE, hematoxylin and eosin; Clt, control; DN16, day 16 of the estrous cycle; DP13, day 13 of pregnancy; DP16, day 16 of pregnancy; DP25, day 25 of pregnancy.

The C3 protein was located in the subcapsular sinuses and lymph sinuses, but there was almost no immunostaining in lymphoid nodules and medullary cords (Fig. 3). The staining intensities for C3 protein were 0, 1+, 1+, 2+ and 0 for the negative control, the lymph nodes from day 16 of the estrous cycle and lymph nodes from days 13, 16 and 25 of pregnancy, respectively (Fig. 3).

## Discussion

The present study demonstrated that *C1q* mRNA and protein levels were increased during early pregnancy, and peaked at day 16 of gestation in the maternal lymph node. C1q is a complex glycoprotein with a C-terminal globular head region and an N-terminal collagen-like tail that mediates a variety of immunoregulatory functions (26). C1q participates in feto-maternal tolerance, trophoblast migration and spiral artery remodeling, and the transcription factor PU.1 regulates decidual C1q expression during early pregnancy in humans (27). Complement C1q can modulate the functions of immune and non-immune cells, and its deficiency and dysregulation result in preeclampsia, missed abortion, miscarriage or spontaneous loss (28). Paternal deficiency of complement component C1q results in a preeclampsia-like pregnancy, and wild-type female mice exhibit renal dysfunction, fetal growth restriction and reduced placental efficiency during mid- and late gestation (29). Upregulation of anti-C1q antibody levels and thyroid-stimulating hormone levels are associated with to autoimmune thyroid disorders during pregnancy in women (30). Mouse C1q is located in various tissues, including lymph nodes (31). Therefore, it is hypothesized that the upregulation of C1q in the maternal lymph node may be important for embryo implantation during early pregnancy in ewes.

The present study revealed that C1r was upregulated in the maternal lymph node at day 25 of gestation. As a modular serine protease, C1r is the autoactivating component of the C1 complex during activation of the next complement components (32). The complement C1r subcomponent protein exists in the maternal serum during pregnancy and can be quantified using isotype tagging in humans (33). Amnion tissue explants and amnion-derived epithelial cells synthesize C1r, which indicates that the amnion synthesizes complement C1r (34). C1q is responsible for the prevention of pregnancy loss, and the immune functions of C1q are mediated by autoactivation of C1r during pregnancy (35). There is an upregulation of the *C1R* gene in peripheral blood polymorphonuclear cells, suggesting that *C1R* is implicated in the early immune response to conceptus presence during the pre-attachment period of pregnancy in heifers (36). Therefore, the upregulation of C1r in maternal lymph nodes at day 25 of pregnancy may be associated with embryonic development in ewes.

The results of the present study demonstrated that early pregnancy induced the expression of C1s in the maternal lymph node, with a peak at day 13 of gestation. The complement component C1 complex is composed of target-recognition subcomponent C1q and modular proteases C1r and C1s, and C1s executes the catalytic function of the C1 complex to cleave C2 and C4(37). C1s is present in the circulating immune complexes during the first trimester of normal pregnancy, and the C1s level decreases during the following weeks of gestation in women (38). C1s is detectable in the placenta, and IFN-γ stimulates the synthesis of C1s in chorion-derived cells (39). The IFN-γ protein is downregulated in bovine peripheral blood mononuclear cells during early pregnancy (40), which may be associated with the decline in C1s, and the loss of immune response to allograft fetus. Complement C1s protein is elevated in maternal plasma of the women with preeclampsia, and is implicated in the remodeling process of the spiral arteries before the manifestation of clinical disease (41). C1s protein is significantly enhanced in the plasma of women carrying Turner syndrome fetuses compared with pregnant women with normal fetuses in the second trimester of pregnancy (42). Therefore, the upregulation of C1s at day 13 of gestation may be associated with initiating implantation, and the downregulation of C1s in maternal lymph nodes at day 25 of gestation may be helpful for pregnancy maintenance in ewes.

The results of the current study also revealed that *C2* mRNA and protein levels were upregulated in the maternal lymph node at day 16 of gestation. Deficiency of C2 leads to autoimmunity, and C2 protein participates in both the classical and lectin pathways of the complement cascade, though C2 is not required for activation of the complement system by the classical or lectin pathway (43). C2 does not participate in activation of the complement system on trophoblastic basement membranes through the classical pathway of complement activation, suggesting that C2 may not be associated with materno-fetal communication during normal human pregnancy (44). C2 deficiency and systemic lupus erythematosus lead to thrombocytopenia and renal abnormalities during the first trimester of pregnancy (45). Interferon stimulates the synthesis of C2 in human monocytes *in vitro* (46). IFNT exerts its effects on maternal lymph nodes (6). IFNT (Protein X) and additional proteins are detected between days 14 and 21, and are produced by conceptus trophoblasts in sheep as previously reported (47). It is suggested that the peak of C2 expression at day 16 of pregnancy in the current study may have been associated with the effects of IFNT, which may not be associated with the activation of complement pathways in the lymph nodes of ewes.

In the present study, C3 peaked in maternal lymph nodes at day 16 of gestation, and then decreased at day 25 of gestation. Component C3 acts as a point of convergence of activation pathways to amplify the complement response, and helps to coordinate downstream immune responses (48). A high level of maternal serum C3 in the first trimester is associated with an increased risk of preterm birth, which can be used for the early diagnosis and prognosis of preterm birth in pregnant women (49). C3 is implicated in the development of preeclampsia through bioinformatics-based identification (50). The serum concentration of C3 is elevated in women with preeclampsia compared with normal pregnant women (51). Human lymph nodes, peripheral blood leucocytes and monocytes can synthesize C3 using an *in vitro* culture technique (52). It is suggested that the decline of C3 in maternal lymph nodes at day 25 of pregnancy may be required for pregnancy maintenance in sheep.

In the present study, *C4a* mRNA and protein were only expressed in maternal lymph nodes at day 25 of pregnancy. C4a is an isoform of C4, and plays key roles in innate immune surveillance, cellular activation and endothelial permeability (53). C4a protein expression is lower in JAR cells under hypoxic conditions compared with normoxic conditions, indicating that preeclampsia is associated with low C4a and hypoxia (54). Maternal plasma C4a concentrations are determined by enzyme-linked immunoassay, and there is a lower C4a level in women with gestational diabetes compared with women with normal glucose tolerance at the time of term delivery (55). A low concentration of maternal plasma C4a is associated with preeclampsia and small-for-gestational age fetuses during pregnancy (56). Pregnant patients with primary anti-phospholipid syndrome or undifferentiated connective tissue disease have significantly lower levels of serum C4 compared with healthy women in each trimester (57). A low amount of *C4* mRNA can be detected in normal human lymph nodes using slot blot hybridization (58). Therefore, it is hypothesized that the upregulation of C4a in maternal lymph nodes at day 25 of pregnancy may be beneficial for successful pregnancy in ewes.

The results of the present study demonstrated that *C5b* mRNA and protein levels were upregulated in maternal lymph nodes during early gestation compared with day 16 of the estrous cycle. C5 protein cleaves into C5a and C5b, and C5b participates in the formation of the membrane attack complex (MAC) associated with C6, C7, C8 and C9(59). C5 convertase cleaves C5 to C5b to result in C5b-9 assembly as the MAC pore on the cell surface (60). C5b-9 is detectable in all placentae, and localized in the surface of syncytiotrophoblasts, intervillous fibrin and decidual vessels (61), which contribute to placental formation. There is an increase in C5b-9 staining within villous trophoblasts of placentas from normal controls compared with patients with preeclampsia in humans (62). In addition, a lack of C5 is associated with embryonic death in Crry-deficient mice, which suggests that C5 plays a key role in preventing embryonic lethality during early pregnancy (63). Therefore, it is reasonable to hypothesize that the upregulation of C5b in maternal lymph nodes may be helpful for pregnancy maintenance during early pregnancy.

The present study demonstrated that early pregnancy induced the expression of *C9* mRNA and protein in maternal lymph nodes at day 16 of gestation. C9 is the final component of the MAC, and the only component of the assembly (64). MACs are involved in pore formation in the plasma membrane of target cells, which is associated with innate and adaptive immune responses (65). Serum C9 levels are significantly higher in healthy pregnant women compared with in non-pregnant women (66). A Japanese woman with C9 deficiency suffered three mid-trimester miscarriages and one early spontaneous miscarriage, suggesting that C9 deficiency is a potential cause of undiagnosed recurrent miscarriage (67). The deposition of C9 is increased in placentae with preeclampsia compared with normal tissues, which is associated with the chorionic villus immunopathology of preeclampsia in humans (68). C9 deposition increases at the implantation sites in pregnant mice with fetal loss (69). C9 concentration is enhanced in the umbilical cord blood of term infants, which is associated with its role in immunity in prematurity (70). Therefore, it is suggested that the upregulation of C9 in maternal lymph nodes at day 16 of pregnancy may be associated with placentation, and the downregulation of C9 at day 25 of pregnancy may be beneficial for pregnancy maintenance in sheep.

Lymph enters the convex system through afferent lymphatic vessels with several branched sinus systems, including subcapsular sinuses, and flows into the blood circulation through efferent lymphatic vessels, including lymph sinuses (71). The immunohistochemistry results of the present study indicated that the immunostaining for C3 protein was located in the subcapsular sinuses and lymph sinuses. Component C3 regulates its receptor expression in B cells from the spleen and lymph nodes, which is implicated in innate and adaptive immune responses in mice (72). Therefore, it was suggested that the downregulation of C3 in the maternal lymph node may be involved in immune tolerance of the maternal lymph node at day 25 of pregnancy in sheep.

In summary, the expression levels of C1q, C1s and C5b were increased during early pregnancy, and the expression levels of C1r and C4a were increased at day 25 of pregnancy only. There were peaks in the expression levels of C2 and C9 at day 16 of pregnancy. C3 was downregulated at day 25 of pregnancy, and C3 protein was located in the subcapsular sinuses and lymph sinuses in the maternal lymph nodes. Therefore, the expression profiles of complement components were changed, indicating that complement pathways may be involved in regulating immune responses of the maternal lymph node during early pregnancy in sheep.

## Figures and Tables

**Table I:** Primers used for reverse transcription-quantitative PCR.

Gene	Primer	Sequence (5'-3')	Size, bp	Accession numbers
*C1qA*	Forward	CAGGAGAACGTGTACCAGAGCAAC	122	XM_012152629.2
	Reverse	CTCCGAGAGGACCTGATGGACAG		
*C1r*	Forward	CCCAGACTACCGCCAGGAAGAG	109	XM_012175492.2
	Reverse	TGGGAGGCAGATTGGCAGGAG		
*C1s*	Forward	CCTGGCAAGTCTTCTTCTCGAACC	130	XM_004006917.4
	Reverse	ACCACTGAGGAGGACCCAACATAC		
*C2*	Forward	CCACCAATCCCATCCAGCAGAAG	95	XM_027958809.1
	Reverse	GGCGTCCAGGAGCAGGTAGAG		
*C3*	Forward	CGCCACCAGCAGACTATAACGATC	105	XM_027969774.1
	Reverse	AGCAGCCTTGACCTCCACCTC		
*C4α*	Forward	TTCAGGACAGGTGGTGAGAGGATC	167	XM_027958803.1
	Reverse	GGAGGAGATGGAGGCGACAGAG		
*C5*	Forward	GCTACGCTGGTGTTACTCTGGATC	157	XM_004003966.3
	Reverse	GCAGACATGACCTCGCCTATAAGC		
*C9*	Forward	GCCGCAACAGAGTGGTGGAAG	138	XM_004017026.3
	Reverse	TGCCATCCCTAACTCGGTCACAG		
*GAPDH*	Forward	GGGTCATCATCTCTGCACCT	176	NM_001190390.1
	Reverse	GGTCATAAGTCCCTCCACGA		

## Data Availability

The datasets used and/or analyzed during the current study are available from the corresponding author on reasonable request.
